# Evaluation of Integrin Glycovariants as Biomarkers of Metastasis, Invasion, and Therapy Stratification in Head and Neck Squamous Cell Carcinoma

**DOI:** 10.1002/cam4.70717

**Published:** 2025-04-27

**Authors:** Erica Routila, Sadie Salminen, Randa Mahran, Mervi Toriseva, Heikki Irjala, Eeva Haapio, Eero Kytö, Sami Ventelä, Kim Pettersson, Johannes Routila, Kamlesh Gidwani, Janne Leivo

**Affiliations:** ^1^ Department of Life Technologies University of Turku Turku Finland; ^2^ InFLAMES Research Flagship University of Turku Turku Finland; ^3^ FICAN West Cancer Centre Turku Finland; ^4^ Institute of Biomedicine, University of Turku Turku Finland; ^5^ Department for Otorhinolaryngology – Head and Neck Surgery University of Turku and Turku University Hospital Turku Finland; ^6^ Turku Bioscience Centre University of Turku and Åbo Akademi University Turku Finland

**Keywords:** glycosylation, HNSCC, integrin, lectin, metastasis, therapy response

## Abstract

**Background:**

Integrin glycosylation is one mechanism regulating the invasion and metastasis of malignant tumors. Little information exists about integrin glycosylation in head and neck squamous cell carcinoma (HNSCC). In this study, we evaluated the glycosylation of integrins in HNSCC tumor and serum samples.

**Methods:**

Intraoperative fresh tumor and normal tissue samples and blood samples were collected from HNSCC patients (*N* = 24). Lectin‐bioaffinity assays using six nanoparticle‐bound lectins were used to evaluate the glycosylation of integrins ITGA2, ITGA3, ITGA5, ITGA6, ITGB1, and ITGB4. Associations with metastasis, therapy response, and clinical factors were analyzed.

**Results:**

Glycosylation profiles of the integrins were relatively similar. High intratumoral ITGB1–WFL results were associated with high T class, whereas none of the integrin glycovariant assays provided significant resolution in the detection of nodal metastasis. While the serum integrin glycovariant levels were low overall, serum ITGA2–UEA offered significant resolution in both radiotherapy response prediction and cancer recurrence prognostication.

**Conclusions:**

We demonstrate that while integrin glycovariants are abundant in HNSCC tumors and ITGB1‐WFL was associated with invasiveness, integrin glycovariants do not directly correlate with metastatic behavior. Further, serum ITGA2–UEA appeared as a potential radioresponse biomarker.

## Graphical abstract

There are scarce data about integrin glycosylation in head and neck squamous cell carcinoma. In this study, glycosylation of ITGA2, ITGA3, ITGA5, ITGA6, ITGB1, and ITGB4 was investigated in intraoperative tissue samples using lectin‐bioaffinity assays. While integrin glycovariants are abundant in HNSCC tumors and ITGB1‐WFL was associated with invasiveness, integrin glycovariants did not directly correlate with metastatic behavior.

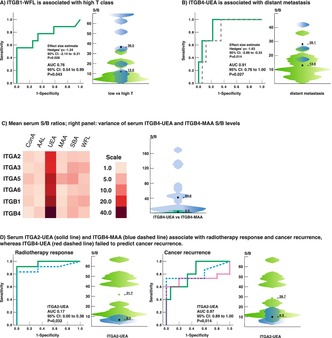

## 1 Introduction

Cancer mortality is strongly related to invasive behavior and especially to metastasis, which represents the most significant hallmarks of cancer. There is a significant variation in the ability of tumors to invade adjacent normal tissue and send metastasis [1]. In addition, cancers such as head and neck squamous cell carcinoma (HNSCC) tend to metastasize along the lymphatic system, whereas other tumor types are more prone to sending distant metastases through the bloodstream [2]. While metastatic behavior may develop over time, HNSCC metastasis is often associated with early stage tumors. Approximately 40% of HNSCC tumors present with lymph node metastasis, which is associated with significantly impaired survival [3].

Integrin (ITG) family proteins have well‐defined functions in the cellular adhesion processes, which can also facilitate the formation of a pre‐metastatic niche, promote the survival of circulating tumor cells, and colonization of metastasis [1, 4]. Controversially, the differential expression of specific ITGs in tumors is associated with oncogenic signaling and metastatic behavior, while the expression of some ITG family members reduces the metastatic tendency. In a recent cell‐line study, integrin β4 (ITGB4) targeting immunotherapy was used to counteract the xenograft potential of carcinoma cells [5]. Surprisingly, relatively few studies have explored the biomarker potential of ITGs in the detection of the metastatic spread of cancer. In one of them, ITGB1 activation by cancer‐associated fibroblasts was recently linked with lymph node metastasis in oral HNSCC [6]. In another example, several ITGs were implicated in local invasiveness and metastasis in papillary thyroid carcinoma [7]. ITG αvβ5 and ITG β6, on the other hand, have been reported to be overexpressed in metastatic tumors of lung cancer and cholangiocarcinoma, respectively [8, 9]. Importantly, in HNSCC, high intratumoral expression of ITGA3 has been linked to lymphatic metastasis and high expression of ITGB4 to hematogenous distant metastasis [10].

Glycosylation is a ubiquitous posttranslational metabolic feature of proteins, where different sugar compounds are bound to the protein structure in an enzymatic process. Aberrations in the glycosylation process of cancer have been observed in virtually all tumor types and can lead to altered specificity and function of proteins expressed by the tumor cells. Altered glycosylation affects the regulatory steps in cancer development and progression, therefore, making the glycan changes an attractive diagnostic and therapeutic target [11]. Glycan structures such as sialyl LewisX (SLe^X^), β1,6‐GlcNAc‐branching N‐glycans, and O‐GlcNAc have previously been linked with the metastatic behavior of cancer [12, 13]. N‐glycans carried by ITGs are required in the ITG–matrix interaction, and accordingly, overexpression of ITGs carrying β1,6GlcNAc‐branching N‐glycans has been linked with cancer cell invasiveness and metastatic behavior [14, 15]. Further, aberration of terminal α2,6‐sialylation of ITGs may alter the metastatic potential of cancer [16]. Importantly, relatively few papers have explored ITG glycosylation in HNSCC, and the majority of them utilize only cell lines instead of patient samples. In one paper, highly fucosylated N‐glycans in ITGB1 and ITGA6 were reported in an oral cancer cell line and were found to be linked with tumorigenesis [17].

The special clinical problem in HNSCC therapy selection is related to difficulties in predicting patient outcomes after surgical treatment due to occult metastatic behavior and field cancerization as well as radiotherapy due to either refusal of chemosensitization or poor radiosensitivity [18]. In this paper, we explored the aberrant glycosylation patterns of different ITGs in HNSCC, with a lectin‐bioaffinity assay developed at our unit [19, 20], allowing for the detection of protein‐specific glycosylations (Figure S1). Further, we evaluate the predictive performance of ITG glycovariant biomarkers to indicate metastatic behavior and invasiveness in prospectively recruited HNSCC patients (*N* = 24). Furthermore, the associations between therapy resistance and ITG glycovariant expression in HNSCC patient tissue and blood samples are investigated.

## 2 Materials and Methods

### 2.1 Cell Lines

Two patient‐derived HNSCC cell lines, UT‐SCC‐14 and UT‐SCC‐60B, were used as previously described [21, 22]. In short, the cells were cultured in Dulbecco's Modified Eagle's Medium (DMEM), with 10% fetal calf serum (FCS), glutamine, and antibiotics (penicillin and streptomycin), and were tested negative for mycoplasma (MycoAlert Mycoplasma Detection Kit, Lonza, Walkersville, ND, USA) and harvested by trypsinization before pelleting. The cell lysates and cell culture media were screened with lectin‐bioaffinity assays.

### 2.2 HNSCC Patient Material

The patient cohort (*N* = 24) was prospectively collected from newly diagnosed HNSCC patients in the southwestern Finland regional tertiary referral center of Turku University Hospital during 2020 and 2021. All the patients received treatment with curative intent and were alive after the follow‐up period (median follow‐up time 12.6 months). Patient informed consent was acquired, and tumor and adjacent normal tissue biopsy samples and blood samples were collected according to ethical approval by the Ethics Committee of the Hospital District of Southwest Finland (Dnro 166/1801/2015). The blood samples were collected in the morning of surgery. Patient characteristics covering treatment and follow‐up data were collected prospectively. The study was conducted in accordance with the Declaration of Helsinki.

### 2.3 Tissue Homogenization

The samples from HNSCC tumor and adjacent normal tissue were homogenized as described previously [21] for further use in lectin‐bioaffinity assays. In short, the tissue samples were homogenized in RIPA buffer (radioimmunoprecipitation assay buffer, 150 mM NaCl, 50 mM Tris, 5 mg/mL sodiumdeoxycholate, 1 mg/mL sodium dodecyl sulphate, 1% NP‐40 [nonionic polyoxyethylene 40]) with 10 μL/mL Halt Protease and Phosphatase Inhibitor Cocktail (Thermo Fisher Scientific) in tissue‐size‐adjusted volume: for tissue pieces smaller than 4 mg, 1 mL RIPA per 3.25 mg tissue sample was used; for pieces < 15 mg, 1 mL RIPA per 7.5 mg sample was used; and for pieces > 15 mg, 1 mL RIPA per 15 mg sample was used. The dilution factors were later reversed in lectin assays. After homogenization with Ultra‐turrax tissue homogenizer (IKA‐Werke GmbH & Co. KG) for 30 s on ice, the samples were kept on ice for 1 h with occasional vortexing. The supernatant from centrifugation at 10,000 *g* for 15 min at +4°C was stored at −70°C. The protein concentration was checked with BCA Protein Assay Kit (Sigma‐Aldrich, USA) according to the manufacturer's instructions.

### 2.4 Preparation of Capture Antibodies and Lectin‐Nanoparticle Tracers

The capture antibodies and lectin‐nanoparticle tracers for lectin assays were prepared as previously described [21]. In short, six monoclonal mouse anti‐human ITG antibodies, namely, anti‐ITG α2 (ITGA2/CD49b, clone HAS3), ITG α3 (ITGA3/CD49c, clone IA3), ITG α5 (ITGA5/CD49e, clone 238307), ITG α6 (ITGA6/CD49f, clone MP4F10), ITG β1 (ITGB1/CD29, clone P5D2), and ITG β4 (ITGB4/CD104, clone 422325) from R&D Systems (Abingdon, UK), were biotinylated with a 40‐fold molar excess of biotin isothiocyanate (BITC) (University of Turku, Finland) in 50 mM NaHCO_3_ (pH 9.8) in a final volume of 200 μL for 4 h, RT, dark. The biotinylated mAbs were purified by NAP‐5 and NAP‐10 gel filtration columns (GE Healthcare, Schenectady, NY, USA) with 50 mmol/L Tris–HCl (pH 7.75), containing 150 mmol/L NaCl and 0.5 g/L NaN_3_, and stored in 1 g/L BSA at +4°C.

The lectin‐nanoparticle tracers were prepared as previously [21, 23], by conjugating 17 lectins (Table 1) purchased from Vector Laboratories (Burlingame, CA, USA) or glycan‐specific Mab provided by Fujirebio Diagnostics AB (Göteborg, Sweden) on Fluoro‐Max polystyrene nanoparticles (diameter 95 nm) (Seradyn Inc., Indianapolis, IN, USA). Briefly, the carboxyl groups on Eu‐NPs were activated by 8 mM N‐hydroxysulfosuccinimide (NHS) and 1.3 mM 1‐ethyl‐3‐(3′dimethylaminopropyl) carbodiimide (EDC) at RT, by vortexing for 15 min. The lectins were coupled to activated Eu‐NPs by vortexing for 30 min, then changing the pH to 8.0 using 0.5 M carbonate buffer, and vortexing further for 30 min. Remaining free carboxyl groups on Eu‐NPs were blocked by 1% BSA while vortexing for 30 min. The lectin‐NPs were purified thrice with 25 mM Tris, 150 mM NaCL, and 0.1% NaN_3_ pH 7.8 before storage in 10 mM Tris–HCl (pH 7.8), 1 g/L BSA, and 0.1 g/L NaN_3_ at +4°C. Before use, the lectin‐NPs were vortexed for 30 min to disperse aggregates.

**TABLE 1:** Lectin panel with glycan‐binding specificities.

Abbreviation	Name	Lectin source	Carbohydrate‐binding specificity
UEA	*Ulex europaeus* agglutinin I	*Ulex europaeus*	α‐linked fucose
DC‐SIGN	Dendritic cell‐specific ICAM‐3‐grabbing nonintegrin, CD209	Mammalian	Mannose, nonsialylated Lewis antigens
MGL	Macrophage galactose lectin	Mammalian	Terminal α‐ or β‐linked GalNAc
MBL	Mannose‐binding lectin	Mammalian	Mannose
C192 Mab	C192	Mouse	CA19.9/Sialyl Lewis a
WGA	Wheat germ agglutinin	*Wheat germ*	GlcNAc
TJA II	Trichosanthes japonica agglutinin II	*Trichosanthes japonica*	Fucose, lactose
AAL	*Aleuria aurantia* lectin	*Aleuria aurantia lectin*	α‐1‐6‐fucose
CON A	Concanavalin A	*Canavalia ensiformis*	α‐d‐mannose, α‐d‐glucose
Gal‐3	Galectin‐3	Mammalian	β galactose
SBA	Soybean agglutinin	*Glycine max*	Terminal α‐ or β‐linked GalNAc, galactose
HPA	*Helix pomatia* agglutinin	*Helix pomatia*	GalNAc
Jacalin	Jacalin	*Artocarpus integrifolia*	Galβ (1‐3)GalNAc
VVL	*Vicia villosa* lectin	*Vicia villosa*	Terminal α‐ or β‐linked GalNAc
RCA	*Ricinus communis* agglutinin	*Ricinus communis*	Galactose, lactose
Gal‐7	Galectin‐7	Mammalian	Galactose, LacNAc
MAA	*Maackia amurensis* agglutinin	*Maackia amurensis*	Siaα2‐3Galβ1‐4GlcNAc
WFL	*Wisteria floribunda* lectin	*Wisteria floribunda*	GalNAc

### 2.5 ITG Glycovariant Detection With Lectin‐Bioaffinity Assay

To identify glycovariants of ITGA3, ITGA6, and ITGB4 in HNSCC cell lines, and glycovariants of ITGA2, ITGA3, ITGA5, ITGA6, ITGB1, and ITGB4 in HNSCC tissue and serum, sandwich‐type lectin‐bioaffinity assays were used as previously described [21]. In brief, 50 ng/25 μL/well biotinylated capture antibody in Red assay buffer (Kaivogen Oy, Turku, Finland) was bound on a Kaivogen Yellow streptavidin‐coated 96‐well microtiter plate at RT for 1 h. After washing the plate twice with Kaivogen Wash buffer, a 1:500 dilution of cell lysate, a 1:2 dilution of cell culture media, a 1:15 or 1:7.5 or 1:3.25 dilution of tissue lysate, or a 1:10 dilution of serum was added and incubated at RT, slow shaking, for 1 h. After washing two times, 1 × 10^7^/25 μL lectin‐NPs were added and incubated at RT, ss, for 1.5 h. Finally, after washing six times, time‐resolved fluorescence (*λ*ex = 340 nm and *λ*em = 615 nm) was measured with Hidex Sense (Hidex Oy, Turku, Finland). To determine the specific signal or signal‐to‐background (S/B) ratio from each sample, the background noise was excluded by dividing the average signal detected from three parallel sample wells by the average signal from three parallel blank wells containing assay buffer without any sample material.

### 2.6 Statistical Analysis

All the data from cell line and HNSCC patient sample screening were analyzed in SPSS 28 software (SPSS, IBM), together with patient characteristics and follow‐up information. The expression levels in tumor versus normal tissue samples and serum samples were compared with a paired *t*‐test. The Kendall rank correlation coefficient was used to evaluate the correlation between assays with the standard interpretation of the correlation strength, where tau‐b (*τ*_*b*_) values ranging from 0.0 to 0.2 are considered very weak, values from 0.2 to 0.4 weak, values from 0.4 to 0.6 moderate, values from 0.6 to 0.8 strong, and values above 0.8 very strong, with negative values indicating inverse correlation. Throughout, 95% confidence intervals were used. The significance was tested with two‐tailed tests, and *p*‐values less than 0.05 were considered significant. The association of glycovariant expression with clinicopathological variables and treatment response was evaluated with independent *t*‐tests. Hedges' *γ* was used to assess the effect size, with 95% confidence intervals. Further ROC analysis was performed on all significant results, except for the age variable, which was further checked in linear regression analysis.

## 3 Results

### 3.1 ITG Glycovariant Assays in Patient‐Derived UT‐SCC Cell Lines

As a proof of concept and for assay optimization purposes, the lectin‐bioaffinity assays specifically measuring various glycovariants of three ITGs, namely, ITGA3, ITGA6, and ITGB4, were first tested in two patient‐derived cell lines UT‐SCC14 and UT‐SCC‐60B. With the data from cell lysate screening, the three most promising plant lectins (ConA, UEA, SBA), two mammalian lectins (DC‐SIGN, MBL), and a glycan‐specific Mab (C192) were chosen for further screening of their respective cell media.

**FIGURE 1:**
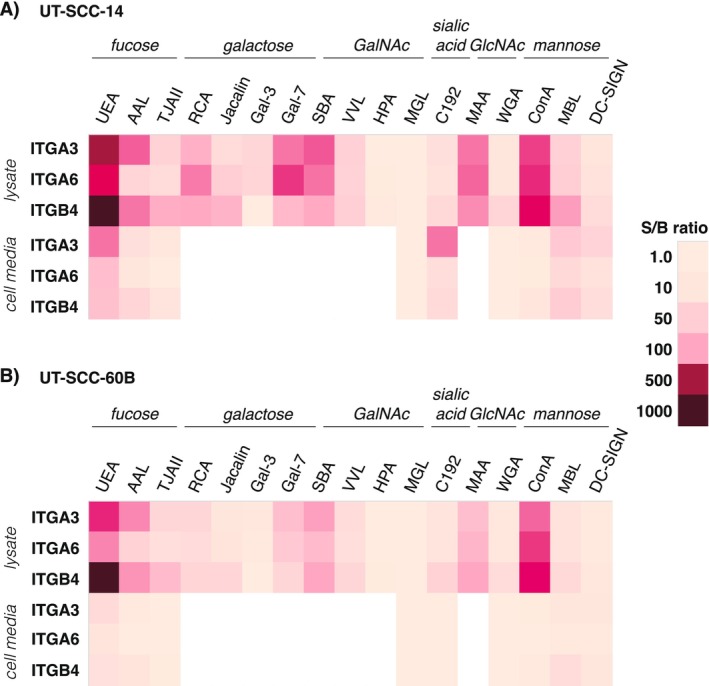
Integrin glycovariant screening of (A) UT‐SCC 14 and (B) UT‐SCC 60B cell lines.

There were significant differences in glycovariant expression in the two UT‐SCC cell lines. Most importantly, the previously cancer‐associated UEA lectin [19, 24] was consistently associated with the highest S/B ratios, ITGB4–UEA being the best antibody–lectin pair (Figure 1). Also, ConA, SBA, AAL, MAA, and Gal‐7 lectins were associated with elevated S/B ratios in both cell lines with all three ITG antibodies. On average, twofold S/B ratios were detected in the UT‐SCC‐14 cell line compared to UT‐SCC 60B with the majority of lectins. The expression levels of ITG glycovariants between cell lysates and cell culture media were compared with a condensed lectin panel (Figure 1). As expected, the S/B levels of ITGs and their glycovariants were significantly from 40‐fold to 40,000‐fold, lower in cell culture media than in cell lysates. In contrast to cell lysates, in cell culture media, ConA was associated with very low S/B ratios, but C192 glycan‐binding protein was associated with elevated S/B ratios.

Considering the cell‐line screening results and at the same time ensuring a wide coverage of different glycosylation modifications, six lectins with the highest S/B ratios, namely, UEA, ConA, SBA, MAA, AAL, and VVL, were chosen for the next step of HNSCC tissue sample screening. VVL, however, was substituted with another GalNAc‐specific lectin, WFL, due to unavailability. For tissue lysate screening, three additional ITG capture antibodies, namely, ITGA2, ITGA5, and ITGB1, were included.

### 3.2 Diverse ITG Glycovariant Expression Across Normal and Cancer Tissues

**FIGURE 2:**
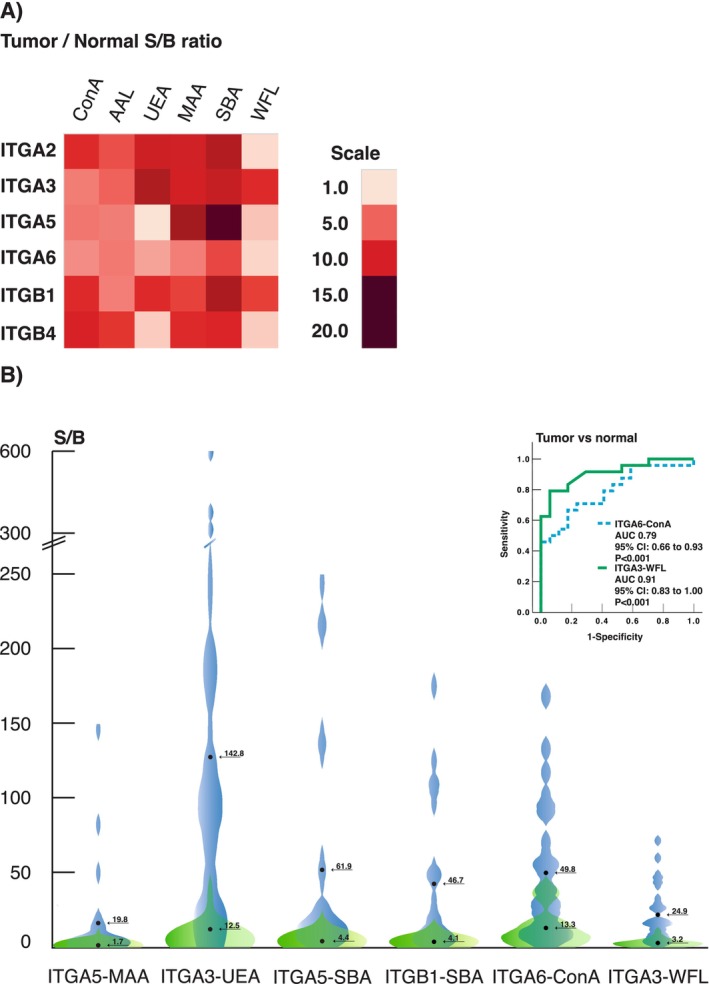
(A) Integrin glycovariant screening from HNSCC tumor versus normal tissue lysates (*N* = 24) as a ratio of S/B ratios. (B) Mean S/B ratios of tumor and normal tissue samples of ITGA5–MAA, ITGA3–UEA, ITGA5–SBA, and ITGB1–SBA, which offered very high tumor versus normal S/B ratios, as well as ITGA6–ConA and ITGA3–WFL, which also performed well in ROC analysis (shown above the violin plots).

In the next step, the expression levels of ITG glycovariants in tumor and adjacent normal tissues from 24 newly recruited HNSCC patients (Table 2) were measured and compared using a paired *t*‐test. The S/B ratios of tumor tissue samples were between 1.0 and 1759 (average 46.5, median 15.1). The adjacent normal tissues showed lower S/B ratios than tumor tissues, with an average S/B of 8.1 and a median S/B of 3.8. Significantly increased ratios between tumor and normal S/B were observed, especially in SBA and MAA assays across all six ITGs, and in ConA and UEA assays with three ITGs (Figure 2A). The smallest S/B ratios were seen in WFL assays across most ITGs and in ITGA6 assays across all lectins. The largest S/B ratios between tumor and normal tissues were observed in ITGA5–SBA, ITGA5–MAA, ITGA3–UEA, and ITGB1–SBA, each demonstrating a large overlap between normal and tumor tissues (Figure 2B). The largest mean differences between tumor and normal tissues were observed in UEA assays ITGA2–UEA, ITGA3–UEA, and ITGB1–UEA. The largest effect sizes were estimated in ITGA6–ConA (*γ* = 1.09; 95% CI 0.49 to 1.67, *p* < 0.001) and ITGA3–WFL (*γ* = 0.98; 95% CI 0.40 to 1.53, *p* < 0.001) assays (Table S1). In ROC analysis, especially ITGA3–WFL demonstrated remarkable performance in discriminating between tumor and normal tissues (Figure 2B).

**TABLE 2:** Characteristics of the patient cohort.

	*n*	%
Gender		
Male	14	58
Female	10	42
Age at diagnosis		
< 70	13	54
> 70	11	46
Smoking		
> 20 pack years	16	67
< 20 pack years	8	33
Alcohol consumption		
Yes	10	42
No	14	58
Primary tumor site		
Oral cavity	16	67
Oropharynx	2	8
Larynx	4	17
Hypopharynx	1	4
Other	1	4
T class		
T0–2	14	58
T3–4	10	42
N class		
N0	16	67
N+	8	33
M class		
M0	21	88
M+	3	13
Recidive during follow‐up		
Yes	5	21
No	19	79
Treatment type		
Surgery only	5	21
RT ± surgery	9	38
CRT ± surgery	10	42

**FIGURE 3:**
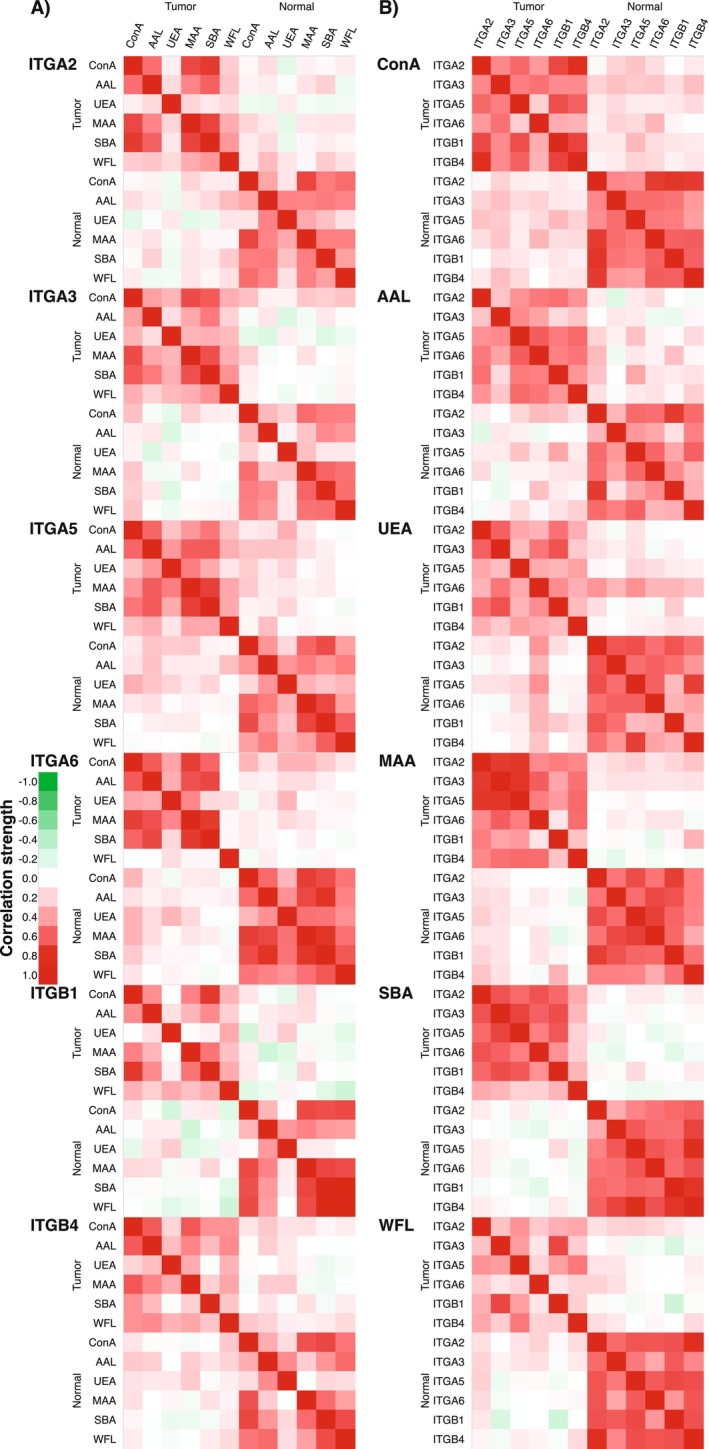
The correlation of integrin glycovariant expression in tumor versus tumor, tumor versus normal, and normal versus normal tissue lysates from HNSCC patients (*N* = 24): (A) between six lectins and (B) between six integrins.

Further, the correlation of ITG–lectin assays between tumor and normal tissue lysates was analyzed with the Kendall rank correlation coefficient (Figure 3, Table S2). Between tumor tissue and normal adjacent tissue, the correlation of glycovariants was very weak to weak, with the only exceptions being the moderate correlation between intratumoral ITGA6–UEA, ITGA2–UEA, and ITGA5–UEA from normal adjacent tissues.

### 3.3 Correlation Between Glycovariant Assays

The correlation between ITG–lectin assays within the tumor tissue sample group and within the normal tissue sample group varied from moderate to very strong (Figure 3, Table S2). On average, stronger correlations were seen, especially when comparing assays that have common lectin but different ITG captures. The strongest correlations were observed between ConA, MAA, and SBA assays. For example, the glycovariants of all six ITGs recognized by ConA shared a strong correlation. The weakest correlations were observed between UEA and all other lectins. The most combinations having strong correlations were seen in ITGA6 assays, especially within the normal tissue group. However, ITGA6–WFL, with lower signal‐to‐background ratios than others, correlates very weakly, if at all, with all other ITGA6 assays in tumor tissue. Further, all WFL assays had only weak to moderate correlations within the tumor tissue group. From the six lectins screened, SBA resulted in the highest number of assays with a strong correlation in tumor and normal tissue groups.

### 3.4 High Glycovariant Expression Was Related to Metastatic and Invasive Behaviors

To rule out any potential confounder bias, the association between ITG glycovariants and key clinicopathological variables and comorbidities was evaluated by the independent *t*‐test (Table S3). No association was observed between the glycovariant assays and patient age or sex. Neither current alcohol, tobacco use, or alcohol exposure history was significantly associated with the glycovariant assays. However, smoking history of at least 20 pack‐years was associated with intratumoral ITGA5–ConA and ITGB4–SBA. Several lectin assays were also found to be significantly associated with potentially confounding comorbidities. Interestingly, ITGA2 and ITGB1 assays with AAL and UEA lectins were significantly associated with cardiovascular disease in adjacent macroscopically normal tissue but not in tumor tissue.

**FIGURE 4:**
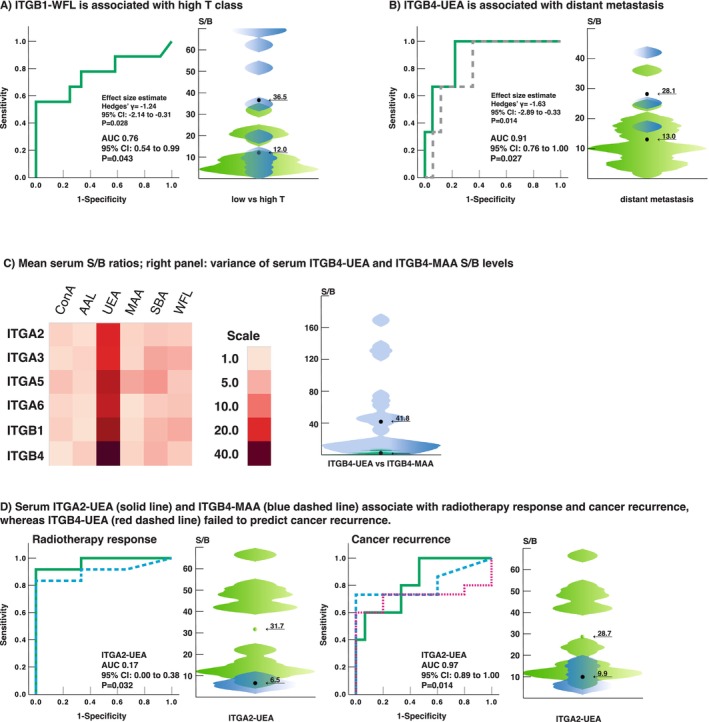
(A) Intratumoral ITGB1–WFL was associated with high T class, and (B) intratumoral ITGB4‐UEA was associated with distant metastasis. (C) In HNSCC patient sera, UEA assays produced significant signals, whereas results of other assays were much lower. (D) Serum ITGA2–UEA (green solid line) and ITGB4–MAA (blue dashed line) associated with radiotherapy response and cancer recurrence, whereas ITGB4–UEA failed to predict cancer recurrence (red dashed line).

After evaluating the independence of the glycovariant assays from the potential confounders, the association between ITG glycovariant expression and high T class, nodal positivity, and distant metastasis was analyzed. For significant results, further ROC analysis was performed. High T class was associated with high intratumoral ITGB1–WFL (Figure 4A), whereas nodal positivity was not significantly associated with any lectin assay. Interestingly, low ITGB1–WFL was significantly associated with the presence of chronic pulmonary disease. Distant metastasis was significantly associated with intratumoral ITGB4–UEA (Figure 4B). However, the rare occurrence of distant metastasis makes this result unreliable.

### 3.5 Low Glycovariant Expression in Blood Samples

The ITG glycovariants were screened also from serum samples (*N* = 24) of the HNSCC patients, and the data were compared with tumor tissue and adjacent normal tissue screenings. The average signal levels of ITG glycovariants in serum were modest (average S/B 7.6, median S/B 3.0), with the exception of UEA assays (average 30.3, median 29.6) and especially ITGB4–UEA (Figure 4C). While the variance of ITGB4–UEA was large (Figure 4C), importantly, the signal levels of ITGB4–UEA did not significantly differ from the other UEA assays, whereas there was a significantly lower signal level in each of the assays using other lectins (paired *t*‐test, data not shown). At best, only a moderate correlation between tumor tissue and serum or between adjacent normal tissue and serum was observed (Table S4). The highest correlation was seen between tumor and serum sample groups in ITGA5–WFL and in ITGA3–WFL.

For analysis of the potential confounding factors, the association of key clinicopathological variables and comorbidities with glycovariant expression in serum was evaluated. No definite association was observed with patient sex or age, nor current alcohol use. While there were moderate significant correlations between age and several WFL assays, these resulted in very weak and nonsignificant linear regression coefficients. However, current tobacco use, long‐term alcohol or tobacco use, diabetes mellitus type 2, and chronic pulmonary disease were all associated with at least one ITG glycovariant in serum (Table S3). Serum ITGA2–UEA levels showed a significant association with high T classification, which was not confirmed in ROC analysis (Figure 4A; dashed line).

### 3.6 The Predictive Potential of Lectin Assays in Treatment Response

Finally, the association between glycovariant expression and HNSCC recurrence or response to radiotherapy was analyzed to assess the predictive potential of the lectin assays. Several lectin assays from tumor tissue showed a significant association with tumor recurrence or radiotherapy response in an independent *t*‐test (Table S3), but further exposure to ROC analysis excluded all the assay candidates (data not shown). Somewhat surprisingly, a low signal level of serum ITGB4–UEA, which demonstrated the highest signal levels among the serum assays (cf. Figure 4C), was associated with tumor recurrence, while the association was not confirmed in ROC analysis. However, interestingly, low signal levels in two lectin assays from serum, namely, ITGA2–UEA and ITGB4–MAA, were significantly associated with both tumor recurrence and poor radiotherapy response in ROC analysis as well (Figure 4D). Importantly, a high signal level of serum ITGA2–UEA was significantly associated with both chronic pulmonary disease and positive alcohol history, which are both markers of poor prognosis. Regarding serum ITGB4–MAA, as the test produced overall very modest S/B levels (cf. Figure 4C), the association is unlikely to offer practical predictive resolution. On the contrary, the S/B levels of the serum ITGA2–UEA assay were among the highest in serum lectin assays (Figure 4C).

## 4 Discussion

Although the role of ITGs in both HNSCC and other cancers has been studied, with consideration focused on the role of ITGs in invasion and metastasis [1, 17], only a few publications have addressed their glycosylation changes, let alone the predictive potential of ITG glycovariants [25]. This is all the more surprising given that ITGs are transmembrane proteins with abundant glycosylation sites and are involved in multiple cancer dissemination‐promoting steps as well as cellular signaling [1]. In fact, to our knowledge, our study is the first to elucidate glycosylation changes of ITGs in a prospectively collected panel of simultaneous samples from HNSCC tumor tissue, normal tissue, and serum. Importantly, with research focusing on de‐escalation strategies in HNSCC, the prediction of radioresistance or tumor recurrence using molecular biomarkers is of utmost importance [26, 27].

The lectin‐bioaffinity assay, which was developed at our unit and has been widely applied to solve various diagnostic problems of different solid tumors [19, 20, 23, 24, 28, 29], allows the detection of low‐abundance glycovariants of a specific protein from tissue samples and liquid biopsies such as serum. The assay utilizes protein‐specific antibodies and glycan‐specific, nanoparticle‐conjugated lectins, resulting in excellent assay specificity and sensitivity [23, 28]. In our previous studies, ITGA3 assays have shown potential for the detection of bladder and ovarian cancers [19, 20, 30]. In this study, we focused on six ITG proteins, which have previously been implicated in metastasis and invasion. Of those, ITGA2 has been associated with field carcinogenesis, a hallmark of HNSCC [31]. ITGA3 has been included in several prognostic gene signature models [32, 33]. ITGA5 glycosylation is linked to the regulation of cell migration [34]. ITGA6 functions as an HPV receptor and has been linked to therapy resistance in HNSCC, making it an especially intriguing target in HNSCC [35, 36] In the cancer genome atlas (TCGA) dataset of HNSCC patients, ITGA3, ITGA5, and ITGA6 all show prognostic resolution [37]. ITGB1 is related to stemness and coexpressed with stem cell markers such as OCT4 and CD44 in tumor cells, whereas its prognostic role remains unclear [38, 39]. ITGB4 overexpression has been demonstrated in HNSCC, and recently, in a mouse model, ITGB4 immunotherapy was successfully used to reduce tumor growth and metastasis [5].

In previous studies, high fucosylation of various proteins has been reported in HNSCC cell lines, tissue samples, and patient sera, and a linkage to therapy resistance has been implied [17, 40, 41, 42, 43]. In accordance, we observed high expression of fucosylated ITGA3, ITGA6, and ITGB4 recognized by UEA lectin in cell lines and in sera of HNSCC patients, whereas in tissue samples, high fucosylation was restricted to three of the six investigated ITGs, namely, ITGA2, ITGA3, and ITGB1. While there was high fucosylation of these ITGs, the variability of the assay signals demonstrates heterogeneity between individual tumors. Interestingly, AAL lectin with slightly different fucose binding specificity [44] did not indicate significantly increased glycosylation of ITGs, and a correlation between UEA and AAL assays was observed in only ITGA5 and ITGA6 assays. Significantly, while the ITGB4–UEA assay resulted in the highest S/B ratio in UT‐SCC cell lysates and in serum samples from HNSCC patients, the S/B ratios of the ITGB4–UEA assay were among the lowest in tumor and normal adjacent tissues. Such distribution of expression may well be related to the production of extracellular vesicles targeting the bloodstream and potentially increasing the metastatic potential of the tumor [45]. Indeed, in cell media, the UEA assays produced the highest signals, which were, however, much lower than the corresponding cell lysate results. The association with extracellular vesicles would be the most plausible explanation, as high tumor ITGB4–UEA expression was related to distant metastasis. However, the expression level of serum ITGB4–UEA was not associated with metastasis.

Most ITG glycovariants were more abundant in tumor than in adjacent normal tissues, suggesting a broad glycosylation pattern across proteins in HNSCC. However, the lack of inverse correlation patterns between normal and tumor tissue samples may be associated with a tendency of the ITG glycovariants to leak outside the tumor perimeter. In addition, high variability in the glycovariant assay signals between individual tumors demonstrates the heterogeneity of HNSCC tumors. Between tumor tissue and adjacent normal tissue, a significant difference was observed in SBA and MAA assays across all six ITGs, with broad glycosylation specificities covering galactose, GalNAc, sialic acid, and GlcNAc. Mannose‐binding ConA assays yielded high S/B ratios with ITGA2, ITGB1, and ITGB4. There were strong correlations within tumor tissue between ConA, MAA, and SBA assays, which lack a common glycan specificity [46]. Interestingly, mannose‐sensitive ITGA6–ConA performed relatively well in differentiating tumor from normal tissue, while the overall expression of ITGA6 glycovariants was low. Taken together, our findings do not support the previous suggestion of highly fucosylated ITGA6 playing a part in HNSCC aggressiveness [17].

Remarkable differences between normal and tumor tissues in GalNAc‐specific WFL assays were observed in ITGA3 and ITGB1. Strikingly, ITGA6–WFL showed a very weak correlation with all other ITGA6 assays in tumor tissue. In ITGA6–WFL, the signal‐to‐background levels were lower than in other ITGA6 assays, which suggests that ITGA6 glycovariants with a GalNAc–glycan structure are not common in HNSCC tumor tissue. While the signal differences of the WFL assays between normal and tumor tissues were relatively low as compared to other lectin assays, suggesting only minor changes in GalNAc structures of these six ITGs, the ITGA3–WFL assay resulted in high discrimination between tumor and normal tissues. Stemness‐related ITGB1 has been linked to local aggressiveness as well as lymph node and distant metastasis in HNSCC [38, 47], whereas the prognostic value of ITGB1 immunohistochemistry remains unclear [39]. Its expression has also been investigated in radiotherapy response prediction [48]. Interestingly, despite the stemness implications of ITGB1, we found its glycosylation profile to be highly concordant with other ITGs. However, high intratumoral ITGB1–WFL was associated with high T class, making it an attractive biomarker candidate for tumor invasiveness. In our recent report, we identified WFL assays of stemness‐related OCT4 and MET to have an association with high T class [21]. Thus, it would seem plausible that there is a WFL‐identifiable stemness‐related regulation in the local invasive behavior of HNSCC. Interestingly, however, no correlation between ITGB1–WFL and WFL assays of other stemness‐associated ITGs ITGB4 and ITGA6 was observed.

To our best knowledge, the predictive potential of lectin assays in treatment response and for tumor recurrence of HNSCC has previously been analyzed in only a single study originating from our laboratory [21]. In the present study, low signal levels of ITGA2–UEA and ITGB4–MAA assays from serum were significantly associated with both tumor recurrence and negative radiotherapy response. However, due to low S/B levels in serum ITGB4–MAA, the predictive potential of this assay as such is very limited. With further optimization of the assay conditions, some improvement might be achievable for ITGB4–MAA performance. The ITGA2–UEA assay, on the other hand, had one of the highest S/B levels in serum lectin assays. Therefore, ITGA2–UEA is suggested to be a potential predictive biomarker for HNSCC radiotherapy response and tumor recurrence, but naturally, further studies including validation by functional analysis would be needed to confirm its role as a biomarker.

Limitations of our study are primarily related to the limited sample size and heterogeneous sample population, keeping in mind that this study provides preliminary insight into ITG glycosylation in HNSCC and warrants further validation in larger patient cohorts. However, such shortcomings have been subdued to the best of our ability through a robust analytical framework and intense interaction between our clinical collaborators. Definite conclusions cannot naturally be drawn from our limited sample size, especially concerning the occurrence of distant metastasis, which is a rare yet devastating event. One limitation is related to the selection of ITG proteins, because in a recent report, glycosylation of ITGAV was associated with nodal metastasis and ITGB6 was associated with local invasiveness [49]. In our study, no significant association was seen between lectin assays and nodal positivity. Since ITG proteins participate in multiple functions in both normal and cancer cells, it is likely that any association with metastasis would also be diluted by the association with local invasive behavior. As a curious side note, ITGA2 and ITGB1 assays with AAL and UEA lectins were significantly associated with cardiovascular disease in adjacent macroscopically normal tissue, but not in tumor tissue. Such findings emphasize the need for careful consideration of potential confounding factors when planning biomarker studies and evaluating their importance, especially when the sample matrix is not directly related to the cancer tissue [50]. As ITG glycovariant assays between serum and tumor tissues or serum and normal tissues correlated only moderately, any conclusions from these results must be interpreted with caution. Regarding ITG glycovariant detection, blood proved to be a challenging sample matrix, possibly indicative of dilution of ITG glycovariants with increasing distance from the primary tumor. Despite the fact that ITGs are key adhesion molecules on tumor‐derived EVs, which are known to spread to blood circulation, with the methods used in this study, it cannot be ascertained that the observed ITG glycovariants in serum originated from tumor tissues [30].

In conclusion, our results indicate that in genetically and behaviorally diverse cancers such as HNSCC, ITG glycovariants could be built into frameworks of clinical decision‐making as additional tools rather than function as independent therapy‐guiding biomarkers. In this line, ITGB1–WFL was associated with local invasion. Importantly, the increase in the expression of promising glycovariants in both metastatic and locally invasive tumors indicates that while being a biologically important phenomenon, ITG upregulation is not a specific event related to a single clinically interpretative process but rather represents a workhorse of malignant transformation. While serum ITGA2–UEA appeared as a potential biomarker for radiotherapy response, the biological rationale for this association could not be confirmed by this study. Nevertheless, our study is the first to explore the aberrant ITG glycosylation simultaneously in both tissue and blood of HNSCC patients. Thus, our results provide meaningful insight into the regulatory mechanisms of HNSCC and outline a structure for future studies.

## Author Contributions

**Erica Routila:** conceptualization (equal), data curation (lead), formal analysis (lead), funding acquisition (supporting), investigation (lead), methodology (lead), validation (lead), visualization (lead), writing – original draft (lead), writing – review and editing (equal). **Sadie Salminen:** data curation (supporting), investigation (equal), methodology (supporting), validation (supporting), writing – review and editing (supporting). **Randa Mahran:** data curation (supporting), investigation (supporting), methodology (supporting), validation (supporting), writing – review and editing (supporting). **Mervi Toriseva:** methodology (supporting), resources (supporting), writing – review and editing (equal). **Heikki Irjala:** funding acquisition (supporting), project administration (supporting), resources (equal), writing – review and editing (supporting). **Eeva Haapio:** investigation (supporting), writing – review and editing (supporting). **Eero Kytö:** investigation (supporting), writing – review and editing (supporting). **Sami Ventelä:** funding acquisition (supporting), project administration (supporting), resources (equal), writing – review and editing (supporting). **Kim Pettersson:** conceptualization (supporting), funding acquisition (supporting), methodology (supporting), resources (supporting), supervision (supporting), writing – review and editing (supporting). **Johannes Routila:** conceptualization (supporting), data curation (supporting), formal analysis (supporting), investigation (supporting), resources (supporting), writing – review and editing (supporting). **Kamlesh Gidwani:** conceptualization (equal), investigation (supporting), methodology (supporting), project administration (supporting), resources (supporting), supervision (equal), writing – review and editing (supporting). **Janne Leivo:** conceptualization (equal), formal analysis (supporting), funding acquisition (supporting), investigation (supporting), methodology (supporting), project administration (equal), resources (lead), supervision (equal), validation (supporting), writing – review and editing (equal).

## Ethics Statement

The tissue and blood samples were collected according to ethical approval by the Ethics Committee of the Hospital District of Southwest Finland (Dnro 166/1801/2015). The study was conducted in accordance with the Declaration of Helsinki.

## Consent

The patient informed consent was acquired from all the patients participating in this study.

## Conflicts of Interest

The authors declare no conflicts of interest.

## Supporting information


**Figure S1.** Flowchart of the study.

**Table S1.** Paired samples effect sizes for tumor vs normal tissue samples.

**Table S2.** Correlation between expression levels of the investigated protein‐lectin pairs in tumor tissue lysates and normal adjacent tissue lysates.

**Table S3.** Association between the lectin bioaffinity assays and TNM factors, recurrence, radiotherapy response, alcohol and tobacco use, comorbidities, sex and age, in tumor tissue, adjacent normal tissue (N) and serum (S).

**Table S4.** Correlation between tumor tissue, adjacent (normal) tissue and serum.

## Data Availability

Data are available from the corresponding author upon a reasonable request.
