# Supplementary material for: Evaluation of Integrin Glycovariants as Biomarkers of Metastasis, Invasion, and Therapy Stratification in Head and Neck Squamous Cell Carcinoma
Source: Cancer Med. 2025 Apr 27;14(9):e70717. doi: 10.1002/cam4.70717 (PMC12034151; doi:10.1002/cam4.70717)
Supplement: Supplementary file 1 — Figure S1. Flowchart of the study. [file CAM4-14-e70717-s001.pdf]

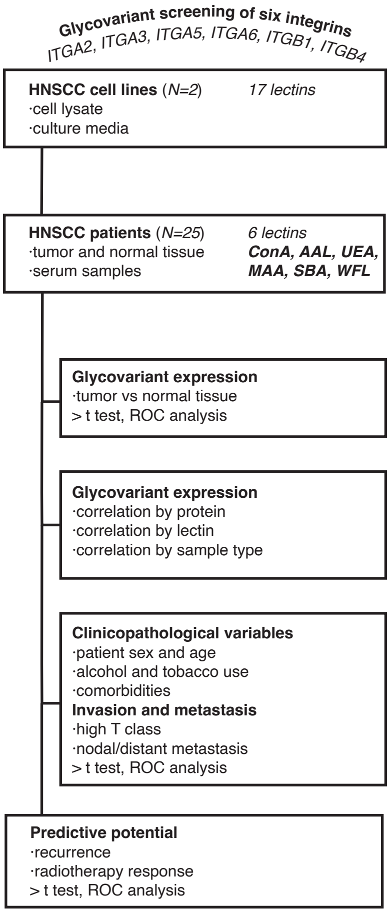

Glycovariant screening of six integrins
ITGA2, ITGA3, ITGA5, ITGA6, ITGB1, ITGB4
HNSCC cell lines (N=2)
·cell lysate
·culture media
17 lectins
HNSCC patients (N=25)
·tumor and normal tissue
·serum samples
6 lectins
ConA, AAL, UEA, MAA, SBA, WFL
Glycovariant expression
·tumor vs normal tissue
> t test, ROC analysis
Glycovariant expression
·correlation by protein
·correlation by lectin
·correlation by sample type
Clinicopathological variables
·patient sex and age
·alcohol and tobacco use
·comorbidities
Invasion and metastasis
·high T class
·nodal/distant metastasis
> t test, ROC analysis
Predictive potential
·recurrence
·radiotherapy response
> t test, ROC analysis
